# Utility of PET-CT in non-small cell lung cancer clinical stage IB-IIA according to AJCC 8th edition staging system: an alternative to invasive mediastinal staging?

**DOI:** 10.3332/ecancer.2021.1250

**Published:** 2021-06-15

**Authors:** Agustin Buero, Domingo J Chimondeguy, Rodolfo Auvieux, Gustavo A Lyons, Leonardo G Pankl, Guillermo Puchulo, Silvia Quadrelli

**Affiliations:** 1 Department of Thoracic Surgery, Buenos Aires British Hospital, Perdriel 74, C1280AEB, Buenos Aires, Argentina; 2 Department of Thoracic Surgery, Austral University Hospital, Av Juan Domingo Perón 1500, B1629AHJ, Buenos Aires, Argentina; 3 Department of Pneumonology, Buenos Aires British Hospital, Perdriel 74, C1280AEB, Buenos Aires, Argentina; a https://orcid.org/0000-0001-5984-3270

**Keywords:** lung cancer, staging, PET-CT, mediastinoscopy, occult N2 disease

## Abstract

**Objective:**

Mediastinal nodal staging in lung cancer is essential to determine treatment strategy and prognosis. There are controversies as to whether a mediastinal negative result in PET-CT may spare the invasive staging of the mediastinum. The main endpoint is to evaluate the negative predictive value (NPV) of PET-CT in non-small cell lung cancer (NSCLC) clinical stage IB-IIA without clinical nodal involvement. The secondary endpoint is to evaluate the prevalence of mediastinal and hilar nodal affection in this population.

**Methods:**

We performed an observational descriptive study from January 2010 to January 2020, including 76 patients with clinical stage IB-IIA, who underwent pulmonary resection with systematic nodal sampling (pre-determined lymph node stations based on tumour location) for primary NSCLC. Clinically, nodal involvement was defined as any lymph node greater than 1 cm in the short axis on a CT or with metabolic uptake greater than 2.5 SUV on PET-CT. The prevalence of nodal metastases was recorded.

**Results:**

Fifty six patients had clinical stage IB and 20 had clinical stage IIA. Mean tumour size was 3.74 ± 0.5 cm. Lobectomy was the resection procedure most frequently performed. Of the 76 patients with clinical N0 by PET-CT who underwent surgical resection, 10 (13.1%) were upstaged to pN1 and none were upstaged to pN2. NPV of PET-CT for overall nodal metastasis was 87% (95% CI: 0.79–0.94). NPV of PET-CT for N2 metastasis was 100%.

**Conclusion:**

PET-CT might be an alternative to invasive mediastinal staging in patients with NSCLC clinical stage IB-IIA who are surgical candidates. Further prospective multi-institutional studies are necessary to verify the external validity of our study.

## Introduction

The accuracy of clinical mediastinal lymph node staging has an implication in the treatment strategy. It is the most relevant factor to determine resectability as the presence of metastasis at this lymph node stations implies an advanced stage (stage IIIA or IIIB). Invasive staging in all patients is controversial. Nowadays is mostly performed in selected patients when there is a high suspicion of lymph node metastasis. According to non-small cell lung cancer (NSCLC) international guidelines [1–4], the criteria include: suspected mediastinal and/or hilar lymphatic involvement in CT (over 1 cm in short axis), hilar and/or mediastinal lymph nodes uptake in PET-CT (SUV over 2.5), central tumours (up to fourth bronchial generation), multiple lung tumours and those greater than 3 cm. In line with international guidelines, PET-CT does not play a decisive role in clinical stages IB-IIA in terms of mediastinal staging, since these patients have a risk factor (tumour size) for N2 and the mediastinum should be explored invasively anyway [1, 5–9]. Although mediastinal staging using PET-CT is superior to CT, many studies show that this method is not flawless. A positive result must be confirmed by histological study due to its low positive predicted value (PPV) [1, 5–8]. Nevertheless, there are controversies whether a negative mediastinal result in PET-CT could spare invasive staging [10–12]. Most authors consider that invasive staging should be performed in patients with risk factors for occult N2 despite a negative result in PET-CT. Hence, according to several international guidelines, invasive staging can only be spared in clinical T1 N0 M0 patients as their reported prevalence of N2 is below 10%. However, in our practice, we have seen that patients who underwent surgery for tumours between 3 and 5 cm without clinical nodal involvement did not have mediastinal nodal metastasis in samples obtained neither by mediastinoscopy nor in surgical specimens. We hypothesise that the absence of clinical node involvement in PET-CT could be an alternative to invasive mediastinal staging in clinical stage IB-IIA. The aim of this study is to evaluate the negative predictive value (NPV) of PET-CT in patients with NSCLC tumours > 3 cm and ≤ 5 cm without clinical nodal involvement and to describe the prevalence of mediastinal (N2) and hilar (N1) metastasis in the study population.

## Methods

This is a descriptive observational study performed from January 2010 to January 2020, where 76 patients were included. Data were collected prospectively and retrospectively from database of two large University Hospitals (Buenos Aires British Hospital and Austral University Hospital). The Institutional Review Committee of the British Hospital of Buenos Aires reviewed and approved this study (CRIHB number: # 802).

### Inclusion criteria

Patients with clinical tumours over 3 cm and less than or equal to 5 cm in diameter without clinical nodal involvement (hilar and mediastinal), who underwent pulmonary resection with systematic nodal sampling for primary lung cancer, were included. These correspond to clinical stages IB and IIA, according to the American Joint Committee on Cancer (AJCC) 8th edition Cancer Staging Manual. Preoperative staging was performed according to the 8th edition of the International Association for the Study of Lung Cancer. All CT scans were done in one of the two hospitals mentioned, with 5 mm thick sections throughout the entire lung during inspiration in the supine position during the injection of intravenous iodinated contrast agent. Data on tumour location, size and standardized uptake value (SUV) were recorded from chest CT and positron emission tomography–computed tomography (PET-CT). Measurements of tumours were performed according to The Fleischner Society: transverse (axial) sections in lung window, unless the maximal dimension lies in a coronal or sagittal plane [13]. Peripheral tumours were defined as those that were located beyond the fourth generation bronchiole, which usually corresponds to the outer two thirds of the lung field (measured as the radial distance from the hilum to the lung periphery). PET-CT scans had been performed to all patients using multidetector CT integrated high-resolution PET-CT scanners after an intravenous injection of Fluorodeoxyglucose F 18 (18F-FDG) (studies from outside institutions were accepted). Clinically, nodal involvement was defined as any lymph node greater than 1 cm in the short axis on a CT or with metabolic uptake greater than 2.5 SUV on PET-CT. Brain magnetic resonance imaging was performed only if the patient had neurological symptoms. Patients must have been suitable for lung surgery: optimal pulmonary function tests, Eastern Cooperative Oncology Group performance status score 0 and 1 and no cardiological contraindication. Non-invasive mediastinal staging (CT and PET-CT) was mandatory within the previous month to lung resective surgery. Having a preoperative invasive mediastinal evaluation was not required as inclusion criteria in the study. When done, it was through cervical mediastinoscopy. Systematic nodal sampling was defined as the biopsy of lymph nodes from pre-determined lymph node stations based on tumour location. Postoperative proven NSCLC histology was required and those with different histology were excluded. Final pathologic stag ing was classified according to the AJCC 8th edition Cancer Staging Manual. Patients with primary NSCLC (cT2a/b) with clinical nodal involvement, previous lung cancer, preoperative chemotherapy-radiotherapy for lung cancer (neoadjuvant treatment) or with PET-CT contraindications were excluded.

### Pulmonary resection and mediastinoscopy

In all patients who underwent a pulmonary resection, systematic nodal sampling with at least four stations was included. A systematic node sample was performed according to tumor location (2R, 4R, 7 and 10R for right-sided tumor’s; 5, 6, 7 and 10L for left-sided tumor’s). Para-oesophageal and pulmonary ligament nodal groups were not explored in all lower lobectomies since it depended on the decision of each surgeon during the surgery. Lobar, segmental and sub-segmental nodal groups were sent together with lung specimen. Taking into account that thoracotomy or left video-assisted thoracoscopic surgery (VATS) cannot satisfactorily explore 2L and 4L nodes (suboptimal intraoperative staging) and that most left-sided tumours did not have mediastinal invasive staging, we decided to analyse whether there were mediastinal recurrences in the follow-up of these patients.

For patients who underwent cervical mediastinoscopy previous to pulmonary resection, our intention was to analyse the following stations: 2R, 4R, 7, 4L and 2L. At least three nodal stations, including stations 4R, 4L and 7, were biopsied.

### Analysis

Statistical analysis was performed using SPSS 13.0 statistical software. The diagnostic value of PET scanning was compared to surgical lymph node assessment by calculating NPV. NPV was calculated as (true negative)/(true negative + false negative). All data are presented as mean +/− standard deviation or counts with percentages.

## Results

Between 2010 and 2020, 76 patients that matched inclusion criteria were selected for the study. There were 42 male and 34 female patients, with a mean age of 67 ± 8.9 years. Fifty six patients belonged to a clinical stage IB and 20 to clinical stage IIA. Mean tumour size was 3.74 ± 0.5 cm (range: 1.96). All patients underwent pulmonary resection with systematic nodal sampling. Lobectomy was the resection procedure most frequently performed (94.7%). The mean lymph node and mean nodal stations evaluated per patient was 10.1 ± 4.7 and 4.3 ± 0.9, respectively (Table 1). Lobar, segmental and sub-segmental nodal groups were not included in the systematic nodal sampling, since they were sent together with lung specimen. Seventeen patients (23.7%) were staged by mediastinoscopy prior to surgery. None of them had mediastinal lymph node affection neither in mediastinoscopy nor in systematic nodal sampling during resective surgery.

Mean follow-up of left-sided tumours was 25.4 ± 15.1 months (median: 24.4 months). Of the 19 patients with left-sided tumours, 3 patients underwent preoperative mediastinoscopy. In the follow-up, one patient developed mediastinal recurrence 8 months after the resective surgery and passed away 10 months after recurrence diagnosis due to COVID-19 pneumonia (19-month total survival). Of the remaining 16 patients who did not undergo mediastinoscopy, none of them had mediastinal recurrence and 3 patients had systemic recurrence (bone, adrenal and contralateral lung) with a mean time of manifestation of 15 ± 9.4 months.

Of the 76 patients with clinical stage N0 by PET-CT who underwent surgical resection, 10 (13.1%) were upstaged to pN1 and none were upstaged to pN2. Multilevel pN1 was detected in one patient (groups 10 and 11). Details of nodal evaluation and upstaging during pulmonary resection are shown in Table 2. NPV of PET-CT for overall nodal metastasis was 87% (95% CI: 0.79–0.94). NPV of PET-CT for N1 was the same as overall nodal metastasis. NPV of PET-CT for N2 metastasis was 100%.

## Discussion

An accurate nodal staging in lung cancer is essential to define treatment strategy and prognosis. International guidelines recommend performing an invasive mediastinal staging in clinical stages IB-IIA due to a high rate of occult N2 (>10%) and a low NPV of PET-CT (<90%) [1–4]. Studies that back up international guidelines were performed using the 7th edition of Classification of Malignant Tumors (TNM) T: tumor; N: nodal; M: metastasis for NSCLC, which has two important differences with the current edition. The first one is that in the previous edition, clinical stages IB-IIA included patients with N1 tumours, whilst the 8th edition does not (Table 3). The second one is that T2 factor differs: in the 7th edition, tumours between 3 and 7 cm were included, while the 8th edition includes tumours between 3 and 5 cm (Table 4). Nowadays, tumours over 5 cm and cN1 are included in stage IIB requiring mediastinal invasive staging due to high incidence of occult N2.

Prevalence of N2 metastasis in patients clinically staged as T2 with N0 by PET or PET-CT has been reported to range from 5.6% to 14.8% [12, 14–17]. As we mentioned above, we believe that these values may be even lower because earlier editions of TNM were used to perform clinical staging. We have not found publications that evaluate exclusively N2 prevalence in such a selected population as our sample. After a thorough analysis of some publications cited previously [14–17], we came to revealing data. Gómez-Caro *et al* [14] describe a prevalence of N2 in cT2 (6th ed. TNM) of 13%. Of 81 patients with cT2, 43 had tumours between 3 and 5 cm (clinical stage IB-IIA of 8th ed. TNM), and of those 4 had occult N2 metastases, with a real prevalence of occult N2 of 9.3%. However, in a later publication in 2012, they report a 14.8% prevalence of N2 for the same tumour size [15]. Lee *et al* [16] describe a prevalence of N2 in cT2 (6th ed. TNM) of 8.7%. Although is under 10%, they described a prevalence of unsuspected N2 for tumours between 2 and 6 cm of 6.45% (6/93). Finally, Gao *et al* [17] describe prevalence of N2 in cT2 of 11.8% including tumours up to 7 cm (7th ed. TNM).

PET-CT is recommended for non-invasive staging of the mediastinum (sensitivity: 80%–90%; specificity: 88%–90%; PPV: 49.3%; NPV: 87%–98%) [1, 5–8]. There is controversy whether negative mediastinal result in PET-CT may spare invasive staging [10–12]. With a PPV close to 50%, for patients with mediastinal uptake is mandatory an invasive approach, due to the high rate of false positive results (inflammatory – infectious disease). On the other hand, with such a NPV, we should ask ourselves if mediastinal invasive staging is necessary for patients without mediastinal uptake. For patients with clinical stage IB-IIA, the NPV of PET-CT described is under 90%. Fernandez *et al* [12] described a NPV for PET-CT of 73.5% for all lymph node metastases (N1 and N2), 79.1% for N1 lymph nodes metastasis and 94.2% for N2 lymph node metastasis. In that publication, 81 of the 90 patients included belonged to clinical stage IB (T2 N0, 6th ed. TNM). From a meta-analysis carried out by Wang *et al* [18], only 130 patients with stage IB (T2N0) NSCLC from two studies were eligible for N2 analysis, yielding a summary NPV of 89% (95% CI: 0.84–0.95). These studies were those of Lee *et al* [16] and Gomez-Caro *et al* [14], with a NPV of 91% and 85%, respectively. Like Fernandez *et al* [12], both used the 6th edition TNM for clinical staging, including greater tumours than 8th TNM edition for clinical T2 tumours. Moreover, we have to consider the limitation of these results, since they may not be much accurate and could be even higher instead if we re-stage these patients with the 8th edition of TNM. It is well known that the greater the tumour, the higher the prevalence of occult N2 and, in consequence, the minor the NPV of the PET-CT [19].

Even though mediastinoscopy is the most accurate method to evaluate mediastinal lymph nodes with high sensitivity and specificity, in patients without nodal uptake in PET-CT it does not offer much benefit and the sensitivity drops to 25%–40% [11, 12, 17]. These diminished percentages partially explain the low prevalence of N2 patients when mediastinal PET-CT is negative. Although the number of patients that underwent mediastinoscopy in our study is too low to achieve significant conclusions, none of them had nodal metastasis in samples obtained neither by mediastinoscopy nor in surgical specimens. Similar results were achieved with the use of endobronchial ultrasound (EBUS) in patients without clinical mediastinal involvement [20]. Despite invasive mediastinal staging methods are of limited utility in this selected population, evidence suggest that unsuspected N2 disease is associated with equivalent 5-year survival compared to cN2 disease if adjuvant therapy is employed [21]. Those results support the use of adjuvant chemotherapy and radiation therapy when confronted with unsuspected N2 disease after surgical resection for stage IIIA-NSCLC [22, 23].

Although the results of the study are flattering and patients with clinical stage IB-IIA could undergo lung surgery avoiding preoperative mediastinal invasive staging, we know that our study has certain limitations: relatively low number of patients, inclusion of patients collected and analysed from a retrospective database (2010 to 2018: 26 (34.2%)), mean number of nodal stations explored does not agree with international recommendations and a suboptimal mediastinal invasive staging in lower lobes and left tumours. In order to diminish the impact of this last factor, we have analysed the presence of mediastinal recurrences in the follow-up of those patients.

## Conclusion

We should reconsider whether patients with clinical stage IB-IIA could undergo pulmonary resection without previous invasive mediastinal staging. International guidelines used earlier TNM’s editions to recommend invasive mediastinal staging in these patients (including patients with tumours over 5 cm and/or N1 involvement) and it is demonstrated that sensitivity of invasive mediastinal staging methods in patients without clinical nodal involvement is very low. In our experience, we believe it might be an alternative since we did not have occult N2 metastasis and the NPV of PET-CT for N2 metastasis was 100%. In order to propose a new recommendation to NSCLC guidelines, further research is needed to validate our results because of our study limitations.

## Conflicts of interest and funding

The authors declare that they have no conflicts of interest or funding.

## Figures and Tables

**Table 1.** Clinical characteristics

Clinical characteristics		
**Variable**		**Mean ± standard derivation or counts with percentages**
Gender		
	Male	43 (56.5%)
	Female	33 (43.5%)
Age (y)		67 ± 8.9
Clinical Stage		
	IB (T2a N0 M0)	56 (73.7%)
	IB (T2b N0 M0)	20 (26.3%)
cT size (cm)		3.74 ± 0.5
Location		
	Central	13 (17.1%)
	Peripheral	63 (82.9%)
Side		
	Right	57 (75%)
	Left	19 (25%)
SUV max		8.2 ± 5
Type of surgery		
	Pneumonectomy	2 (2.7%)
	Bilobectomy	1 (1.3%)
	Lobectomy	72 (94.7%)
	ULL	54
	LLL	18
	Segmentectomy	1 (1.3%)
No. of LND		4.3 ± 0.9
No. of LN		10.1 ± 4.7
Histology		
	Adenocarcinoma	69 (90.8%)
	Squamous cell	7 (9.2%)

*ULL: upper lobe lobectomy; LLL: lower lobe lobectomy; LNS: lymph node stations; LN: lymph node*

**Table 2.** Lymph node evaluation and upstaging during pulmonary resection

Lymph node stations	No. of patients with nodal stations sampled (*n*=76)	No. of patients with nodal metastasis (*n*=10)
2R	30	0
2L	0	0
4R	53	0
4L	0	0
5	18	0
6	15	0
7	67	0
8	6	0
9	9	0
10R	51	3
10L	12	1
Bronchopulmonary	67	7

**Table 3.** Differences between stages IB-IIA in 6^th^, 7^th^ and 8^th^ edition of TNM classification

TNM			
Stage	6^th^ ed.	7^th^ ed.	8^th^ ed.
IB	T2 N0 M0	T2a N0 M0	T2a N0 M0
IIA	T1 N1 M0	T2b N0 M0	T2b N0 M0
		T1 N1 M0	
		T2a N1 M0	
IIB	T2 N1 M0 T3 N0 M0	T2b N1 M0	T1 N1 M0
			T2 N1 M0
			T3 N0 M0

**Table 4.** Differences between T2 tumor size in 6^th^, 7^th^ and 8^th^ edition of T2 classification

T2		
6^th^ ed.	7^th^ ed.	8^th^ ed.
>3 cm	3 - 7 cm	3 - 5 cm
	2a: 3-5 cm	2a: 3-4 cm
	2b: 5-7 cm	2b: 4-5 cm
